# Virtual Diabetes Prevention Program Tailored to Increase Participation of Black and Latino Men: Protocol for a Randomized Controlled Trial

**DOI:** 10.2196/64405

**Published:** 2025-06-24

**Authors:** Earle C Chambers, Elizabeth A Walker, Clyde Schechter, Eric Gil, Terysia Herbert, Katelyn Diaz, Jeffrey Gonzalez

**Affiliations:** 1 Albert Einstein College of Medicine Bronx, NY United States; 2 Yeshiva University Bronx, NY United States

**Keywords:** diabetes prevention, minority populations, men's health, virtual, diabetes, prevention, Black, Latino, randomized controlled trial, RCT, clinic-based, standard care, weight loss, risks, effectiveness, adoption, hypothesize, electronic health record, EHR, academic medical center, health system partner, Power-Up intervention, mixed-gender

## Abstract

**Background:**

Black and Latino men are at increased risk for poor diabetes health outcomes but are underrepresented in lifestyle interventions for weight loss and diabetes prevention. Although relatively few men participate in the National Diabetes Prevention Program (NDPP), it remains the most widely available evidence-based approach to type 2 diabetes prevention in the United States. Thus, an NDPP tailored to Black and Latino men has the potential to address prior limitations of NDPP implementation and reduce gender, racial, and ethnic diabetes disparities. It also provides an opportunity to define a population for targeted outreach and evaluate the reach of our recruitment methods and interventions.

**Objective:**

We tailored the US Centers for Disease Control and Prevention Prevent T2 curriculum for the NDPP for Black and Latino men, called Power-Up*,* and will evaluate its effects in comparison to standard mixed-gender NDPP groups via virtual delivery. The primary aim of the project is to assess the effect of Power-Up versus NDPP on weight loss among men with prediabetes. The secondary aim is to compare the engagement and retention of men with prediabetes in Power-Up versus NDPP. We will also examine the reach of our recruitment methods and engagement in our screening, consenting, and assessment procedures prior to the point of randomization. We hypothesized that men randomized to Power-Up would achieve greater percent weight loss from baseline at 16 weeks (end of Core sessions) and 1 year (end of Maintenance sessions) than men randomized to standard, mixed-gender NDPP. Power-Up is also expected to have better engagement and retention.

**Methods:**

Using the electronic health record (EHR) systems of a large academic medical center and a network of small to medium independent primary care practices throughout New York City, we identified Black and Latino men who met eligibility criteria for NDPP and enrolled them in a randomized controlled trial in which they were assigned 1:1 to receive Power-Up or the standard, mixed-gender NDPP over 1 year via online videoconferencing. Coaches delivering these interventions were trained according to the standards for the NDPP. Power-Up will be delivered by men coaches. Weight will be collected with home-based electronic scales for primary outcome analyses. Engagement will be assessed by session attendance logs.

**Results:**

We identified 11,052 men for outreach based on EHR data, successfully screened 26% of them, consented and enrolled 22% of these, and randomly assigned 48% of consented participants. Primary and secondary outcome analyses will be assessed among randomized men.

**Conclusions:**

This study highlights the effort required to reach and engage Black and Latino men for virtually delivered diabetes prevention programs. Forthcoming trial results for weight loss and engagement will further inform efforts to address disparities in diabetes prevention through tailored programming for Black and Latino men.

**Trial Registration:**

ClinicalTrials.gov NCT04104243; https://clinicaltrials.gov/study/NCT04104243

**International Registered Report Identifier (IRRID):**

DERR1-10.2196/64405

## Introduction

### Background

The prevalence of type 2 diabetes and its risk factors disproportionately affects low-income and racial and ethnic minority populations in the United States [1,2]. This mirrors international evidence showing disparities in diabetes prevalence related to socioeconomic disadvantage and structural racism [3]. In the United States, Black and Latino populations are 2-3 times more likely to die of diabetes-related complications than their White counterparts [2]. In general, more men than women have prediabetes, and fewer men are aware of their condition than women [4]. Men are more likely than women to be hospitalized for long-term complications of diabetes and more than twice as likely as women to have a leg or foot amputated. Compared to women, and compared to other men, men of color have significantly poorer health and higher rates of hospitalization related to diabetes [5]. Latino men are twice as likely to die from diabetes as men identifying as non-Hispanic White, and men identifying as Black are 3 times as likely to need treatment for diabetes-related kidney disease, as compared to non-Hispanic White men [2]. Compared to non-Hispanic White men, men of color are significantly more likely to have a lower limb amputation [6,7]. Despite these striking disparities, men of color are rarely the focus of research, policy, or practice focused on the prevention of diabetes or other chronic diseases [8-10].

The Diabetes Prevention Program randomized trial (DPP; 32% male participants) clearly established the efficacy of lifestyle change for weight loss and type 2 diabetes prevention [11,12]. There have been many efforts to translate the DPP into “real world” settings, but they mostly reach women [13]. Evaluation of the National Diabetes Prevention Program (NDPP) showed that men comprise fewer than 20% of participants [13]. Men of color are particularly underrepresented in NDPPs [13-15]. However, when men do participate, they achieve equivalent or better weight loss than women [13]. Low rates of participation of men of color in the NDPP are consistent with low levels of engagement in other healthy activities and health promotion programs [16,17]. Although the body of literature on men of color and in health programs is still limited [18], researchers attribute low participation in healthful activities to competing priorities that are compounded by the impact of structural, contextual, and life course factors, including discrimination, poverty, and a cycle of limited education and limited employment opportunity [18,19]. Poor engagement of men has been attributed to the framing of existing programs [20,21] and masculine ideals and societal expectations that men be self-reliant, suppress signs of weakness, and avoid seeking help [22,23]. Men-focused programming is a promising method for reaching and engaging men and achieving behavior change [24]. Men-only group-based programming may facilitate behavior change, may increase openness with respect to health concerns, and can act as an incentive for participation [25]. Retaining and tracking the health outcomes of men in these programs is essential to evaluating success and sustainability. Men of color are particularly underrepresented in NDPPs. The Power-Up randomized controlled trial, an adaptation of the NDPP tailored to the needs and preferences of Black and Latino men [26-28], will evaluate weight loss and engagement outcomes for Black and Latino men [26-28].

### Specific Aims

The delivery of Power-Up includes a men-only, virtual, group-based model with men as coaches and an NDPP curriculum designed to be attentive to the concerns and needs of men. Virtual DPP is defined as a DPP program delivered remotely as synchronous classes through a videoconferencing platform. The intervention will be delivered virtually to participants recruited through clinical settings and available in English and Spanish. The primary aim of the project is to assess the effect of Power-Up versus NDPP on weight loss among men with prediabetes. The secondary aim is to compare the engagement and retention of men with prediabetes in Power-Up versus NDPP. We will also examine the reach of our recruitment methods and engagement in our screening, consenting, and assessment procedures prior to the point of randomization. The results of this study will help to fill an important gap in health promotion and diabetes prevention for men, specifically for Black and Latino men.

## Methods

### Study Development

The Power-Up study (ClinicalTrials.gov NCT04104243) was developed in partnership with our study team at Montefiore-Einstein in collaboration with partners at the New York City Department of Health and Mental Hygiene (DOHMH) Primary Care Improvement Project (PCIP). Montefiore is a large hospital system in the Bronx, NY, and the largest provider of health care in Bronx County. The PCIP program is a collection of health care practices throughout New York City that are supported by the DOHMH particularly, as it relates to this study, in the delivery of diabetes prevention and management programs. In 2019 when the Power-Up study was funded, it was designed to be delivered in-person, and the curriculum had been updated from the pilot delivery [28] to be consistent with the new Prevent T2 guidelines. Approximately 6 months into the study development phase of the randomized trial, the first cases of COVID-19 reached New York City, and shortly after the city was shut down. The delivery of DPP, which was a priority of quality health care within our hospital system and through our DOHMH community clinic partners, was eclipsed by the pandemic, which used all available resources. The Bronx bore the heaviest burden of the early wave of the pandemic in New York City, and New York City more generally became the epicenter of the pandemic accounting for a large share of the mortality from the virus. In the months that followed, it became clear that in-person delivery of DPP would not be possible for at least a year, or longer. A decision was made to convert the study to virtual delivery. This process meant the training of coaches in virtual delivery including the use of virtual platforms for both the Power-Up coaches and all coaches delivering the control condition in collaboration with our DOHMH partners and their affiliated clinical partners. It also meant that eligibility criteria had to be changed to include patients who had access to a stable internet connection (although participants could attend via phone) and were comfortable with accessing the Zoom or Microsoft Teams platform. Curriculum materials were shared digitally or on paper via mail delivery, if requested. Another important change is that we modified our original plans for in-person weight collection to remote collection using digital scales. Questionnaire data will be collected remotely (see Survey Questionnaires). These data will be collected at baseline, after the 16th session is offered, and at the end of 12 months (see Survey Questionnaires. The aims of the Power-Up study remain the same and the study became more important as we learned of the increased risk of mortality that COVID-19 disease would have among people with diabetes. Below we report the details of the protocol for the trial in accordance with SPIRIT (Standard Protocol Items: Recommendations for Interventional Trials) [29] reporting guidelines as well as CONSORT-EHEALTH (Consolidated Standards of Reporting Trials of Electronic and Mobile Health Applications and Online Telehealth; V 1.6.1) [30] checklist (Multimedia Appendix 1).

### Setting

Study materials and procedural development took place in our study offices. The recruitment, eligibility screening (Textbox 1), and enrollment procedures were conducted by study staff or prevention outreach specialists located at Montefiore-Einstein and PCIP/DOHMH offices. Data collection from electronic health record (EHR) systems took place at the respective clinical site locations. All engagement data will be collected during NDPP sessions. Survey data will be collected by phone at baseline when randomization takes place and during routine calls from study staff at 16 weeks and 12 months. The weight measurements will be collected via a global system for mobile-enabled weight scales (BodyTrace Inc). In data analysis, missing weights will be managed using multiple imputations from time points near NDPP session delivery from GSM (global system for mobile communication) scale weights, weights extracted from EHR, and self-reported weights.

**Inclusion and exclusion criteria.**

**Inclusion criteria**

- Identified as Hispanic or Latino and or African American or Black as indicated in the electronic health record and later by self-report during screening.
- Identifying as a man via electronic health record initially and later by self-report during screening.
- Age 18 years or older.
- BMI ≥ 25 kg/m^2^ (within the last 6 months) and most recent hemoglobin A_1c_ (HbA_1c_): 5.7%-6.4% (within the last year) or Centers for Disease Control and Prevention prediabetes risk score of 5 or higher.
- Valid residential address and telephone contact information.
- Plan to remain in the New York City area for at least a year or more.
- Received care at a New York City–based Primary Care Improvement Project health care clinic or Montefiore Medical Group within the last 12 months.

**Exclusion criteria**

- Previously diagnosed with diabetes.
- Inability to join most sessions via virtual platform or by phone.
- Unable to participate in either English or Spanish.
- Previous participation in Diabetes Prevention Program.
- Inability to join during session time or day.

### Participant Recruitment

Our recruitment strategies were informed by our experience with the pilot and prior health behavior change and lifestyle intervention studies conducted by our team. The strategies included regular data queries from our clinical sites to identify eligible men for participation. Men who met the initial eligibility criteria based on information available in the EHR (eg, weight, height, and hemoglobin A_1c_ [HbA_1c_]) were called and invited to participate once eligibility criteria were established (see Textbox 1). Future engagement strategies will include monetary incentives for the completion of telephone surveys and the collection of weight data at the initial baseline interview, as well as a 16-week core session and the final maintenance session at 12 months.

At the Montefiore Health System sites, we generated lists of men who (1) met all the following NDPP criteria based on EHR data, at least 18 years old, most recent BMI ≥25 kg/m^2^ (within last year), most recent HbA_1c_ 5.7%-6.4% (within last 12 months); and (2) received care at a Bronx-based Montefiore Health System site in the past 12 months. We contacted eligible men by phone to inform them about the study and to obtain their consent to participate. To be fully eligible, men needed to (1) self-identify as African American, Black, Hispanic, or Latino during telephone screening; (2) have no plans to change their primary care provider or move from their current address in the next year; (3) agree that they are physically able and willing to attend virtual, group-based diabetes prevention classes; (4) complete the baseline survey assessment and be willing to complete follow-up study surveys and procedures for collecting study weights; and (5) provide informed consent by telephone, including consent for study participation, data collection, and random assignment to Power-Up or standard care NDPP.

DOHMH engaged small primary care practices (PCP) that serve predominantly Black and Latino populations to identify eligible individuals receiving care in the last 12 months at a PCIP health care clinic for recruitment into the Power-Up study. After securing an agreement from the PCP, DOHMH facilitators provided on-site technical assistance to participating PCPs to generate a patient report using their EHR systems to apply study eligibility criteria every 6 months. DOHMH facilitators were instructed to generate eClinicalWorks Business Optimizer (eBO) reports at each PCP’s clinical location. These eBO reports use a prewritten query already saved at each practice EHR that identifies individuals that meet study eligibility criteria. The query retrieves a comprehensive set of data for each eligible individual that includes full name, date of birth, date of last office visit, sex, race and ethnicity, preferred language, address, phone number, last HbA_1c_ value and date measured, height, weight, and BMI. Generated reports were transferred to a Microsoft Excel document and securely transmitted to DOHMH data staff. To confirm eligibility criteria, DOHMH data staff reviewed eBO reports and assigned eligible individuals for telephonic outreach.

The Power-Up study was designed to recruit 300 men, with 1:1 random assignment, 150 randomly assigned to the men-only Power-Up condition run by the Einstein study team and another 150 into a mixed-gender NDPP comparison condition run by the PCIP partners. In order to keep the comparison condition ratio of men to women similar to the upper limit of male participation in current NDPP implementation across our partnering health systems, we will monitor the percentage of men assigned or referred to these groups during recruitment and work with our health system partners to refer additional eligible women, through EHR-based searches and outreach to ensure that the percentage of men in standard care NDPP groups does not rise substantially in any one group. We will distribute the randomized men in the comparison condition over 4 mixed-gender groups to reduce the likelihood that the percentage of men in any of those groups would be higher than 33%. Thus, for every Power-Up men-only group there will be 4 mixed-gender groups running concurrently, with 4 times as many women recruited for those groups as men.

### Randomization

Separate randomized sequences of treatment group assignments for English and Spanish language–preferring participants, in balanced blocks of sizes 4 or 8 in random order, were computer-generated using Stata version 16.1 and maintained securely at a remote site by the data analyst (CS), who will have no contact with the participants. Random assignment and final enrollment into the study occurred after telephone screening, obtaining informed consent, and completion of baseline surveys. The data analyst was blinded to condition assignment.

### The Power-Up and Standard NDPP Conditions

The DOHMH provided NDPP master training for all coaches. The first training occurred for one coach in the middle of year 1, before beginning interventions, and a second training was offered to 3 coaches at the end of the second year. The training curriculum included instructions on data collection for all coaches (1/2 day). Men coaches implementing Power-Up received additional training in the adapted curriculum and using a toolbox for men-tailored behavioral approaches (extra 1 1/2 days). Supervision was available by experienced co-investigators to ensure fidelity to the protocol. Additional periodic training was given to coaches to ensure they were up to date with current strategies to administer NDPP. Study staff conducted regular meetings with coaches from both the Power-Up and the standard NDPP conditions to be sure session delivery and all training were consistent between the conditions. All coaches facilitated either the standard Power-Up or NDPP conditions, with no crossover of coaches. Because many of the patient population in our clinical catchment areas are more comfortable communicating in Spanish, we offered both Power-Up and NDPP conditions in Spanish as well as English.

Both Power-Up and the standard NDPP will follow the PREVENT T2 curriculum which emphasizes (1) beginning core sessions with a focus on physical activity, (2) de-emphasizing tracking fat grams, (3) adding a session on heart health, (4) developing more content on replacing sugary drinks with low-calorie drinks, and (5) emphasizing coping strategies. All Power-Up and standard NDPP programs include 2 optional presession orientation classes (ie, session 0) prior to the 16 core sessions delivered over 6 months and 8-10 additional maintenance sessions delivered over the subsequent 6 months for a total of 26 sessions over 1 year. These sessions will discuss logistics and ensure that participants are able to get connected to the platform. The standard NDPP curriculum will be delivered virtually at a standard time and day of the week that is responsive to standard work schedules and will take place typically in the evenings. After the first session, the day of the week and time can be changed to accommodate most of the participant’s schedules. One make-up session will be offered per week for any missed session by participants. The sessions will be delivered in either English or Spanish depending on the preferred language of the participants, which will be determined at the baseline interview. The delivery of sessions will be coordinated so the Power-Up and standard conditions start within at least 2 weeks of each other. Participants will be given the session curriculum digitally or by paper copy mailed out to them upon request. All participants will receive activity, food, and weight logs digitally or by mail. They will also receive digital handouts with strategies that can help with weight loss and calorie tracking. The sessions will be delivered at no charge to participants.

Adaptations to the NDPP curriculum focused on making the program more appealing and motivating to men, more consistent with their priorities, group dynamics, and behaviors, and more likely to affect lifestyle change*.* These adaptations were made to the Prevent T2 curriculum based on our pilot work [26-28] and an updated review of the literature. The Power-Up sessions differ from the standard NDPP in that they will be run by men coaches, and include only participants identifying as men, and the PREVENT T2 curriculum was adapted to focus on men using men-centric vignettes, illustrations, and health-related information. Our goal was to develop an adaptation based on the input of men who participated in the pilot [26-28], that would meet Centers for Disease Control and Prevention (CDC) criteria as a recognized program (Table 1). Recruitment strategies included messaging emphasizing that the program was specifically designed for men and facilitated by men coaches.

**Table 1:** Power-Up curriculum content.

Session	Session topic	Session focus
1	Welcome to Power-Up	This introductory session helps men at risk for diabetes change their lifestyles by moving them from the thinking phase to the action phase.
2	Get Active to Stay Active	This session introduces the concept of getting active.
3	Be Safe, Be Active, Keep on Track	This section provides detailed instructions on how to track your activities and how to exercise safely.
4	Build Your Healthy Plate	This session introduces the concept of healthy eating.
5	Track Your Food	This session provides detailed instructions on how to track the food you eat.
6	Being Active- A Way of Life	This session focuses on how to become more physically active as a lifestyle choice.
7	Find your Tipping Point: Burn more calories than you take in	Teaches participants how to burn more calories than they take in.
8	Eating from Shopping to Cooking	This session focuses on how to become more physically active as a lifestyle choice.
9	You Can Manage Stress	This session teaches participants how to reduce and deal with stress.
10	Four Keys to Healthy Eating Away from Home	Teaches participants how to stay on track with their eating goals at restaurants and social events.
11	Jump Start Your Activity Routine	This session teaches participants how to find time for fitness.
12	Taking Charge of What is Around You	This session teaches participants how to cope with the triggers of unhealthy behaviors.
13	Power-Up Your Heart	This session teaches participants how to keep their hearts healthy.
14	Talk Back to Your Negative Thoughts	This module teaches participants how to replace harmful thoughts with helpful thoughts.
15	Getting Support	This module teaches participants how to get support for their healthy lifestyle.
16	Stay Motivated to Prevent Diabetes	This module helps participants reflect on their progress and helps them to keep making positive changes over the next 6 months.
17 (Maintenance Session)	Staying Active for the Long Haul	This session teaches participants how to start losing weight and to cope with some challenges of staying active.
18 (Maintenance Session)	Take A Fitness Break	This session teaches participants how to overcome barriers to taking fitness breaks.
19 (Maintenance Session)	Staying Active Away from Home	This session teaches participants how to stay on track with their fitness goals when they travel for work or pleasure.
20 (Maintenance Session)	Understanding Carbs	This module gives participants a deeper understanding of carbs.
21 (Maintenance Session)	Have Healthy Food You Enjoy	This module teaches participants how to have healthy food that they enjoy.
22 (Maintenance Session)	Get Enough sleep	This module teaches participants how important sleep is to their lifestyle change.
23 (Maintenance Session)	Get Back on Track, Man!	This module helps participants stay positive and know that although there are obstacles they can get back on track to their goals.
24 (Maintenance Session)	Prevent Diabetes for Life!	This module helps participants to reflect on the past year, seeing how it helps create a plan for the next 6 months.

### Data Collection

**Figure 1:**
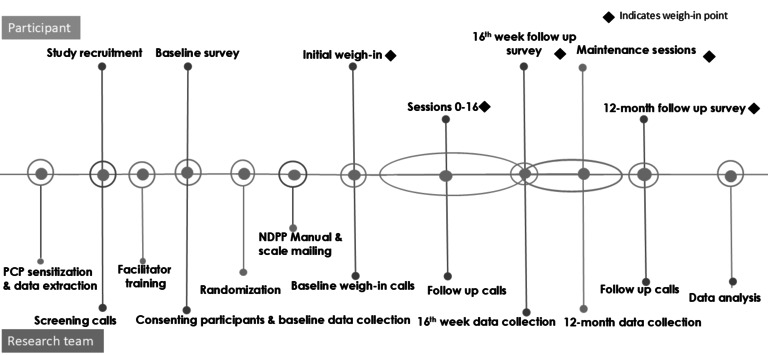
Data collection timeline. EHR: electronic health record; NDPP: National Diabetes Prevention Program.

The data collection timeline is shown in Figure 1.

### Outcome Measures

Weight is the primary outcome measure based on the focus on weight loss as a mechanism for diabetes prevention in the original DPP trial and the compelling evidence linking weight loss of 5%-10% of body weight with clinically significant reductions in diabetes risk [31]. Participants will use study-supplied GSM-enabled scales from Body Trace [32] to measure and transmit their weight to coaches and the study team. To maximize the completeness of these data, incentives will be provided to participants who transmit weight measurement at sessions or provide a self-reported weight. When a weight measurement is not available using these scales at the 16-week and 12-month sessions (primary outcome measurement time points), then a self-reported weight by telephone or by search of EHR on a visit close to the session dates will be used. The primary outcome measure is the percent change in weight at the end of the Core sessions and the end of Maintenance sessions. Study staff and trained coaches will collect and manage all primary outcome data (Figure 1).

### Survey Questionnaires

Questionnaires for psychosocial and behavioral variables will be conducted by telephone by study staff. All questionnaires were translated into Spanish; however, not all were rigorously psychometrically evaluated for language differences. Survey assessment will occur at 3 time points: baseline or enrollment (immediately prior to randomization), at 16 weeks, and 12 months. Table 2 shows the survey domains and time points of questionnaire data collection for the study.

**Table 2:** Survey domains and data collection time points.

Survey domains	Baseline	16-week follow-up	12-month follow-up	Measure
Demographics^a^	✓			Power Up 1.0
Insurance	✓			NYC^b^ CHS^c^ [33]
Housing insecurity	✓		✓	Study of Latino Mobility Study
Housing composition		✓		Affordable Housing as an Obesity Moderating Environment Questionnaire
Social needs+food insecurity	✓		✓	Adapted from Health Leads [34]; Hager et al. [35]
Health history	✓	✓	✓	HCHS/SOL^d^ [36]
Body image perception	✓	✓	✓	National Physical Activity and Weight Loss Survey [37]
Sleep	✓	✓	✓	Pittsburgh Sleep Quality Index [38]
Sedentary behavior	✓	✓	✓	International Physical Activity Questionnaire [38]
Physical activity	✓	✓	✓	NYC CHS [33]
Diet	✓	✓	✓	NYC CHS [33]
Risk perception	✓	✓		Risk Perception Survey-DD [39]
Self-rated health	✓	✓	✓	NHANES^e^ [40]
Depression	✓	✓	✓	Patient Health Questionnaire-8 [41]
Tobacco use	✓			HCHS/SOL [36]
Alcohol	✓			NYC CHS [33]
Neighborhood environment	✓	✓	✓	Power Up 1.0
Discrimination	✓			Everyday Discrimination Scale [42]
Machismo	✓			HCHS/SOL [36]

^a^Age, gender, race and Hispanic ethnicity, education, employment, and income.

^b^NYC: New York City.

^c^CHS: Community Health Survey.

^d^HCHS/SOL: Hispanic Community Health Study/Study of Latinos.

^e^NHANES: National Health and Nutrition Examination Survey.

### Secondary Outcomes: Engagement and Retention

Evaluation of engagement and retention for aim 2 is based on attendance records for Power-Up and standard NDPP sessions electronically collected by trained coaches and monitored by study staff. We will follow standards for NDPP evaluation where engagement is defined as ≥4 core sessions attended and retention is defined as ≥9 sessions attended. We will examine several processes and moderating variables based on survey measures.

### Ethical Considerations

This study was approved by the institutional review board at the Albert Einstein College of Medicine (registration number 2019-10343). All participants provided oral informed consent for screening, which was obtained by the study staff. All data will be stored on a secure, password-protected computer, and identifying information will be removed from all data management and analysis files. If participants meet screening eligibility, they will receive US $40 for completing the initial set of baseline assessments (US $15 for completing the baseline survey and US $25 for providing a baseline study weight), US $25 if they provide a study weight for session 16, and US $25 if they provide a study weight for the final session. They will receive additional payments of US $15 for the completion of the follow-up assessment at 12 months. Participants can provide study weights without session attendance, and participants can receive the full compensation associated with completing that assessment regardless of their attendance. We will monitor and report any protocol deviations for approval by the Albert Einstein College of Medicine institutional review board, and document on ClinicalTrials.gov, as appropriate.

### Data Analysis

The nature of our study intervention makes it difficult, if not impossible, to blind participants and coaches to their study arm; however, we did not directly disclose our hypotheses to them. The data management and analysis protocols we outline below may be modified based on unanticipated data anomalies that must be resolved in some way. To ensure that these decisions are undertaken without bias, our statistician will be given an end-of-study dataset that designates the arms neutrally as group 1 and group 2. Until all data management and analyses are complete, the statistician will not be told which group is Power-Up and which is NDPP. A future study will present descriptive statistics by arm for end-of-study variables including percent weight loss and retention. To identify covariates for inclusion in analyses, we will draw a directed acyclic graph of perceived causal relationships among all variables. After excluding any colliders of the treatment (outcome relationship and variables on a causal path between treatment and outcome), variables other than the treatment itself having an unblocked backdoor path to the outcome will be considered potential confounders and will be included as covariates.

### Specific Aim 1: Percentage Weight Loss

The primary intention-to-treat (ITT) analysis will contrast the percentage weight loss across study arms using mixed-effects linear regression models of 16-week and 12-month percentage weight loss with the study arm as a predictor, and random intercepts at the program group and practice levels. Because of the large sample size and random assignment to groups, it is very unlikely that adjustment for confounding variables will be required. If, however, our descriptive statistics identify variables that differ appreciably between arms and are associated with outcomes, they will be adjusted as covariates. A secondary per-protocol analysis using only those participants who have completed at least 4 sessions will be carried out using models similar to the primary ITT analyses. Because per-protocol analyses do not preserve randomization, we will reanalyze and carefully compare the baseline attributes across arms to seek out confounders to be included as covariates. For these analyses limited to successfully engaged participants, we expect equivalence of Power-Up and Standard NDPP and will interpret these results by examining whether the 95% confidence limits of the difference in percentage weight loss fall within 2.5 percentage points of zero.

### Specific Aim 2: Engagement and Retention

Engagement and retention outcomes will be analyzed with ITT mixed-effects logistic regression models using the same predictors and random intercepts. In all instances, we will report the model-predicted expected values with 95% CIs as well as the expected difference in outcomes with its 95% CI values calculated by the delta method. The *P* values from Wald tests of the null hypothesis of no difference between arms will also be shown.

Missing scale weights will be principally managed using multiple imputations by chained equations. Any unexpected missing engagement and retention data will be dealt with first by excluding cases with missing data from analysis and second by using multiple imputation procedures. If these approaches yield differing outcomes, both will be reported in our outcome paper. For all analyses, we will report the model-predicted expected values with 95% CIs as well as the expected difference in outcomes with its 95% CI calculated by the delta method. The *P* values from Wald tests of the null hypothesis of no difference between arms will also be shown.

### Power Calculations

Our target sample size was 300 men. Power calculations assumed, based on earlier studies, a mean (SD) of 1.5 (SD 4.7) percentage points in percent weight loss, and an intraclass (intraprogram group) correlation of 0.05. Assuming 80% of participants provided complete data for analysis, we would have 90.5% power to detect a 2.5-percentage point difference in weight loss between the study arms, as well as providing 80% power to detect a Cohen *d* of completion and engagement outcomes of 0.21 and 0.18, respectively. Power to detect a 5-percentage point difference in weight loss between the study arms would be >99.9%.

As a worst-case sensitivity analysis, we considered the possibility that the SD of percentage weight loss could be as high as 5.9-percentage points, and the intraclass correlation as high as 0.10. Under these adverse circumstances, we would still retain 81.4% power to detect the 2.5-percentage point difference at the .05 significance level. Power for the engagement and retention outcomes was not appreciably affected by changes in these parameters.

## Results

**Figure 2:**
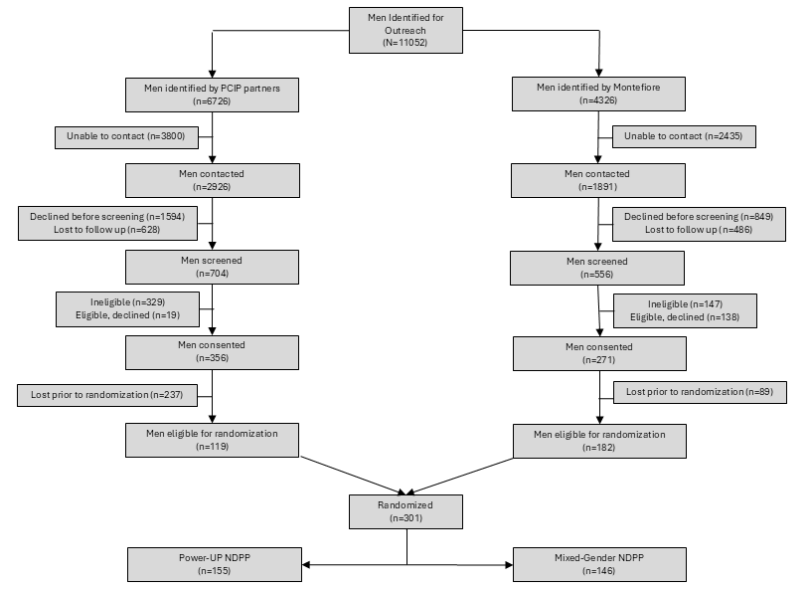
CONSORT (Consolidated Standards of Reporting Trials) diagram for study. NDPP: National Diabetes Prevention Program; PCIP: Primary Care Improvement Project.

Of the 301 men randomized in our study (Figure 2), we identified 11,052 potentially eligible men based on clinical weight and HbA_1c_ information identified in the EHR. Of the men identified, 43.5% (4817/11,052) were contacted and asked to participate in a yearlong NDPP. Of the men contacted to participate, 6.2% (301/4817) agreed to participate and were ultimately randomized.

## Discussion

### Overview

Lifestyle interventions that can prevent type 2 diabetes among men of color are urgently needed to address the disparities in participation for these men in the United States. A prior report of NDPP delivery using data from all CDC-accredited programs showed that men are only 20% of NDPP participants nationwide [13]. However, the reach of NDPP and uptake of the NDPP programs more generally is largely unknown as most studies start measuring engagement at the point of attendance at the first NDPP session [43].

We based our adaptation of the NDPP for Black and Latino men on relevant partnership input, formative quantitative and qualitative results, and lessons learned from a successful pilot study in men with prediabetes [24-26] to enhance the acceptability and potential for maximal weight loss outcomes of the Power-Up intervention tested in the current trial. While we do not yet know the rate of engagement in Power-Up post randomization, we had to identify a population of 11,052 men who were preliminarily eligible based on data recorded in the EHR. Of these, 4817 men were successfully contacted, 2871 were successfully screened, and 628 consented. Of these consented men, 327 did not complete baseline assessment procedures or were otherwise lost to follow-up before they could be randomized. Thus, our primary and secondary outcome analysis sample of 301 men represents only 6% of the men we were able to contact through our extensive telephone outreach efforts and less than 3% of those we identified for contact. Our recruitment experience highlights the substantial resources needed to reach this population. This rate of engagement is low but consistent with previous reports of the high cost of resources needed to recruit eligible participants and deliver NDPP [44]. Studies show that delivery of NDPP in US clinical settings may not be fiscally feasible given the cost of delivery even when reimbursement by Medicare or Medicaid is considered [45-47]. Furthermore, a recent report in poor rural areas of the United States showed that this disparity of uptake in poorer communities may exacerbate diabetes disparities [48]. This is of particular concern in poor urban settings like the Bronx where even with NDPP offered free of charge uptake of the intervention is low [49,50]. Success with targeted engagement of underserved Black and Latino men will likely require significantly more resources related to outreach than prior studies have suggested. Despite these challenges to NDPP delivery, there may be benefits to delivering NDPP tailored for men in that once enrolled in the program their retention rates may be better than mixed-gender approaches.

### Study Implications

The Power-Up study is unique in that the recruitment was conducted from patients identified in the EHR system of clinics and so has a known denominator for recruitment. An analysis using the true denominator facilitated the evaluation of the true reach of our recruitment efforts. Power-Up was designed to leverage the past success of NDPP delivery through the clinical care setting by identifying and recruiting eligible men seen in primary care practices of a large hospital system in the Bronx, NY, and primary care settings supported by the DOHMH. This approach also allowed for the identification of potential participants through the EHR systems facilitating data collection on important risk factors for type 2 diabetes, such as weight and HbA_1c_.

Together with our experience reported here on the reach of our recruitment efforts, trial outcomes will provide important information on the relative benefits of Power-Up as compared to standard, mixed-gender NDPP. Primary outcomes examining differential weight loss and secondary outcomes evaluating differences in engagement will be of interest to various partners involved in paying for promoting, delivering, and participating in the NDPP. These results may also have relevance to other national and international settings where similar lifestyle interventions targeting weight loss and type 2 diabetes prevention are offered in mixed-gender groups with lower-than-expected participation rates for men.

Our study design also has limitations that will need to be considered in the interpretation of the results. First, prediabetes is a condition that is inherently heterogeneous with respect to the risk of developing type 2 diabetes, especially given the range of assessments that can be used to identify it in clinical and research settings. Although our approach to determining eligibility with EHR-extracted data and the NDPP risk score may have identified a heterogeneous population of men with respect to phenotype and risk, these procedures are consistent with current practices for NDPP enrollment in the United States. Thus, reduced rigor around the definition of prediabetes comes with the benefit of resulting in a research design that is more translational and able to address the broader question of the effectiveness of the intervention in a real-world clinical context. We sacrificed additional rigor related to the assessment of our primary weight loss outcomes. Home-based electronic scales are likely to be more vulnerable to errors in measurement and missing data than in-person weight collection. However, in-person collection was not feasible in the context of the early period of the COVID-19 pandemic and the virtual delivery of the intervention. Furthermore, the use of home-based weights also mitigates against biases associated with the collection of weights being dependent on NDPP session attendance. We will have electronic weights on participants, regardless of their attendance, permitting a less biased assessment of effects on weight in ITT analyses. Collection of weights through additional methods when electronic weights are unavailable is a strength of the study design that will allow for sensitivity analyses including estimates for missing data. Finally, our results will be limited in their generalizability to men who are engaged in primary care settings and have access and facility with online videoconferencing. Virtual delivery of Power-Up and NDPP sessions may have also enhanced participation and engagement by removing barriers to in-person attendance for men, particularly during the early phase of the pandemic. Our success with meeting our recruitment target, despite the challenges reported here, is a strength of our study and will ensure that our outcome evaluations are well-powered.

## Appendix

Multimedia Appendix 1 CONSORT-eHEALTH checklist (V 1.6.1).

## Abbreviations

CDC: Centers for Disease Control and Prevention
CONSORT-EHEALTH: Consolidated Standards of Reporting Trials of Electronic and Mobile Health Applications and Online Telehealth
DOHMH: Department of Health and Mental Hygiene
DPP: Diabetes Prevention Program
eBO: eClinicalWorks Business Optimizer
EHR: electronic health record
GSM: global system for mobile communication
HbA_1c_: hemoglobin A_1c_
ITT: intention to treat
NDPP: National Diabetes Prevention Program
PCIP: Primary Care Improvement Project
PCP: primary care practice
SPIRIT: Standard Protocol Items: Recommendations for Interventional Trials

## Data Availability

The datasets generated during and/or analyzed during this study are not publicly available due to the fact that identifiable information is included but a de-identified dataset can be made available from the corresponding author on reasonable request.
